# Impact of the COVID-19 pandemic on adults with Fetal Alcohol Spectrum Disorder: linking immune function to mental health status

**DOI:** 10.3389/fnins.2023.1214100

**Published:** 2023-07-19

**Authors:** Tamara S. Bodnar, Amanda Chao, Parker J. Holman, Linda Ellis, Charlis Raineki, Joanne Weinberg

**Affiliations:** ^1^Department of Cellular and Physiological Sciences, Faculty of Medicine, University of British Columbia, Vancouver, BC, Canada; ^2^Department of Psychology, Brock University, St. Catharines, ON, Canada

**Keywords:** prenatal alcohol exposure (PAE), cytokines, immune system, white blood cells, depression, anxiety

## Abstract

Prenatal alcohol exposure (PAE) is known to cause a variety of cognitive, behavioral, and neurological changes. Importantly, mental health problems are also overrepresented in individuals with Fetal Alcohol Spectrum Disorder (FASD), the group of neurodevelopmental conditions that can occur following PAE. Approximately 90% of individuals with FASD report experiencing mental health problems over their lifespan, compared to approximately 30% in the overall population. Individuals with FASD also display impairments in coping skills and increased vulnerability to stress. Here, we investigated whether the COVID-19 pandemic would have a differential impact on mental health and inflammation-to-mood associations in adults with FASD, compared to unexposed controls (no PAE). We capitalized on our pre-pandemic study examining health and immune function and invited past-participants to enroll in the current study. Participants completed mental health assessments and COVID-related questionnaires by phone. In addition, blood samples collected at baseline (pre-pandemic) were used to probe for inflammation-to-mood associations. Overall, our results indicate that lower SES was predictive of higher coronavirus anxiety scores, with no differences between adults with FASD and controls. In addition, while there were no differences in depression or anxiety measures at baseline (pre-pandemic) or during the pandemic, examination of inflammation-to-mood associations identified differential relationships in adults with FASD compared to unexposed controls. Specifically, there was a positive association between baseline neutrophil counts and both baseline and pandemic mental health scores in unexposed controls only. In addition, for unexposed controls there was also a negative association between baseline interferon-ɣ (IFN-ɣ) and pandemic mental health scores. By contrast, only adults with FASD showed positive associations between baseline interleukin-12p70 (IL-12p70), IL-8, soluble intercellular adhesion molecule-1 (sICAM-1), and soluble vascular cell adhesion molecule-1 (sVCAM-1) and pandemic mental health scores. Taken together, to our knowledge, this study is the first to examine the impact of the pandemic in adults with FASD. And while it may be too soon to predict the long-term effects of the pandemic on mental health, our data suggest that it will be important that future work also takes into account how immune function may be modulating mental health outcomes in this population.

## 1. Introduction

The COVID-19 pandemic has resulted in unprecedented changes to daily life worldwide (Haleem et al., 2020). Public health measures aimed at decreasing the spread of coronavirus, including cancelation of events and social distancing from friends and family, have resulted in unfortunate consequences such as increased loneliness and impaired psychological well-being (Okruszek et al., 2020). It has been established that rates of mental health problems, such as depression and anxiety, increased substantially during the pandemic (Kwong et al., 2021; Hawes et al., 2022; Wettstein et al., 2022). In addition, there are subgroups within the general population known to be especially vulnerable to poor mental health as a result of the COVID-19 pandemic including women (Almeida et al., 2020), people in low-income households (Kwong et al., 2021), and individuals with a previous history of mental health problems (Neelam et al., 2021). Another group shown to be at higher risk are those with neurodevelopmental disorders (NDDs) (Guller et al., 2022). Notably, the majority of this work has been conducted in children, with reasons for the increased risk of mental health problems attributed to changes in routines (e.g., disruptions to schooling and extracurricular activities) and increased fear of infection (Ehrler et al., 2021; Guller et al., 2022). Adults with NDDs, including attention deficit hyperactivity disorder (ADHD) and autism spectrum disorder (ASD), also experienced increased rates of depression and anxiety during the pandemic (Shakeshaft et al., 2023). However, it is important to note that not all of the impacts of the pandemic on mental health were negative for these populations. For example, one study reported that, for adult females with ASD, depressive symptoms *decreased* during the pandemic, potentially due to the reduced demands/opportunities for social interactions (Shakeshaft et al., 2023). Similarly, decreased sensory and social overloading were also reported as positive impacts of the pandemic on mental health for adults with NDDs (Oomen et al., 2021).

Much less is known regarding the impact of the pandemic on mental health in individuals with Fetal Alcohol Spectrum Disorder (FASD), the group of neurodevelopmental conditions that can occur following prenatal alcohol exposure (PAE). Importantly, FASD is the most common neurodevelopmental disorder, with current North American prevalence estimates at approximately 4–7% (Flannigan et al., 2018; May et al., 2021), which is higher than that of ASD, cerebral palsy and down syndrome combined (Cook, 2021). Of relevance, individuals with FASD may be at particularly high risk of impairments to mental wellbeing during the pandemic for reasons including the high rate of existing mental health problems in this population (Pei et al., 2011; Coles et al., 2022), as approximately 90% of individuals with FASD experience a mental health disorder in their lifetime (Famy et al., 1998), as well as an apparent link between lower socioeconomic status (SES) and FASD (Bingol et al., 1987; Abel and Hannigan, 1995; May and Gossage, 2011). Despite the high prevalence of FASD and the added mental health risk factors, very little is known in terms of how the pandemic impacted mental health in these individuals.

Pre-clinical and clinical studies have demonstrated that *in utero* alcohol-exposure has significant negative impacts on immune development and function [reviewed in Bodnar and Weinberg (2013), Reid et al. (2019), Lussier et al. (2021)]. Specifically, children with FASD broadly exhibit increased rates of infection (Johnson et al., 1981; Gauthier et al., 2005) and atopic conditions (Linneberg et al., 2004; Wada et al., 2016), as well as altered immune cell counts (Johnson et al., 1981; Gottesfeld and Abel, 1991; Oleson et al., 1998) and cytokine levels (Ahluwalia et al., 2000; Bodnar et al., 2020). Much less is known regarding immune function in adults with FASD; however, preliminary results from our ongoing research suggest that immune system disruptions persist into adulthood, including alterations in cytokine levels (Raineki et al., 2021; McMahan et al., 2023). Importantly, positive associations between inflammatory cytokines and mood symptoms have been consistently reported (Anisman et al., 2005; Raison et al., 2006; Kohler et al., 2017; Leighton et al., 2018). In addition, pre-clinical and clinical evidence indicates that cytokine increases can precipitate depressive-like symptomology (Reichenberg et al., 2001; Dunn et al., 2005; Liang and Ghany, 2013). While inflammation-to-mood associations have been generally well-established, the specific cytokines involved vary based on factors including the health status and environment of the individual (Rosenblat et al., 2014). For example, psychological stress has generally been shown to increase levels of C-reactive protein (CRP), interleukin (IL)-6, IL-1β, and tumour necrosis factor (TNF)-α in otherwise healthy individuals (Steptoe et al., 2007), whereas for obese individuals, elevated levels of IL-5, IL-12, and interferon-ɣ (IFN-ɣ) have been associated with depression (Schmidt et al., 2014). By contrast, inflammation-to-mood relationships have yet to be explored in individuals with FASD. A better understanding of inflammation-to-mood relationships could highlight opportunities for early-recognition, prevention and treatment of mood disorders (Rosenblat et al., 2014).

In the current study, we examined the impact of the pandemic on mental health in adults with FASD, as well as the link between immune function and mental health outcomes. We capitalized on our ongoing, pre-pandemic study examining health and immune function in adults with FASD, re-contacting study participants and inviting them to participate in the current study to re-examine their mental health during the pandemic. Overall, we hypothesized that mental health would be impaired in adults with FASD as a result of the pandemic and specifically, that higher scores than unexposed controls (no PAE) on anxiety and depression assessments would be identified. In addition, we hypothesized that inflammation-to-mood associations would differ between adults with FASD and unexposed controls due to differential impacts of pandemic-related stress on these two populations. Specifically, we hypothesized that, given the known immune dysregulation in individuals with FASD, the pandemic would act as a stressor such that mood dysregulation would be higher in adults with FASD who had a higher immunological set-point pre-pandemic.

## 2. Materials and methods

### 2.1. Cohort description

Participants were recruited from our ongoing Collaborative Initiative on Fetal Alcohol Spectrum Disorders (CIFASD) study examining health and immune outcomes in adults with FASD, which was paused in March 2020 due to the pandemic. Briefly, for this broader CIFASD study, inclusion criteria included: adults (19+) with a diagnosis of FASD (i.e., Fetal Alcohol Syndrome [FAS], partial FAS [pFAS], Alcohol-Related Birth Defects [ARBD], Alcohol-Related Neurodevelopmental Disorder [ARND] or a related diagnosis) living in British Columbia (BC), Canada. Participants were asked to provide details regarding their diagnosis (i.e., year of diagnosis, clinic/doctor who performed the diagnosis, etc.) and participants who did not have a diagnosis of FASD were unable to participate in the study. Participants with FASD were recruited through social media, advertising at events and conferences related to FASD, and through flyers posted at FASD diagnostic clinics. A matched group of unexposed controls (no PAE) was also recruited from the community through social media, online advertisements, and posting study flyers in libraries, coffee shops and other community locations. Potential participants were excluded if they could not provide informed consent, were currently pregnant or breastfeeding, or had HIV/AIDS.

Of the 46 adults with FASD and 26 unexposed controls who had been recruited and had completed the broader CIFASD study by March 2020, and who had provided consent to be re-contacted, a total of 19 adults with FASD and 17 unexposed controls participated in the current pandemic study, carried out over the phone, from January 2021 – May 2021. This timeframe was recognized by the Public Health Agency of Canada as a peak of COVID infections, with 2,800 - 9,000+ daily cases of COVID-19 documented in BC, representing the largest peak in infections in BC up to that point (Statistics Canada, 2023). Study questionnaires administered pre-pandemic (during in-person testing for our broader CIFASD study) were repeated for the current pandemic study (by phone), with the addition of the COVID-19 assessments.

### 2.2. Demographic information

Questionnaires were used to collect demographic information including race/ethnicity, income, education, employment status, neighborhood characteristics etc. (Table 1). Demographic information was also used to generate a composite measure of socioeconomic status (SES), as has been previously reported (Gilman et al., 2017). Briefly, this SES measure combined information regarding: (1) education (incomplete high school, high school graduate, post-high school education); (2) income relative to the Canadian 2021 low-income cut-off (LICO), adjusted for family size (<LICO, 100–150% of LICO, >150% of LICO); (3) occupation [unemployed, employed – manual/part-time, employed (non-manual/full-time]); (4) neighborhood safety (is it safe to walk alone at night in your neighborhood? [mostly unsafe, somewhat safe, safe]). For each of the four factors, a score of 0, 1, or 2 was assigned, with higher scores, indicating a higher degree of socioeconomic advantage and the score for each of the four factors summed to produce a composite measure of SES (Supplementary Table 1).

**Table 1:** Demographic information.

Variable		Unexposed control (*n* = 17)	FASD (*n* = 19)	*p* value
Age (years)		36.1 ± 3.0	37.2 ± 2.4	0.350^1^
Diagnosis (self-report)	Fetal Alcohol Syndrome (FAS)	–	42.1% (8)	–
	Partial FAS (pFAS)	–	10.5% (2)	
	Alcohol Related Neurodevelopmental Disorder (ARND)	–	5.3% (1)	
	FASD (specific diagnosis unknown)	–	42.1% (8)	
Current gender identity	Man	23.5% (4)	21.1% (4)	0.586^2^
	Woman	76.5% (13)	78.9% (15)	
Ethnicity	Indigenous	11.8% (2)	57.9% (11)**	**0.010**^**3**^
	White	35.3% (6)	26.3% (5)	
	Other ethnicities	52.9% (9)	5.3% (3)*	
Education level	Incomplete high school	5.9% (1)	31.6% (6)	0.107^2^
	Graduated high school	11.8% (2)	26.3% (5)	
	Partial college	11.8% (2)	15.8% (3)	
	College or University graduate	23.5% (4)	15.8% (3)	
	Graduate professional training	47.0% (8)	10.5% (2)	
Employment status	Employed	70.6% (12)	42.1% (8)	0.287^2^
	Full-time student (or vocational training)	0% (0)	5.3% (1)	
	Volunteer work	0% (0)	10.5% (2)	
	Unemployed	29.4% (5)	36.8% (7)	
	Missing	0% (0)	5.3% (1)	
Current income	<$20,000	23.5% (4)	57.9% (11)	0.080^2^
	$20,000–$49,999	23.5% (4)	26.3% (5)	
	$50,000–$99,999	17.6% (3)	5.3% (1)	
	>$100,000	29.4% (5)	5.3% (1)	
	Missing	5.9% (1)	5.3% (1)	

Questionnaires (self-report) were used to collect demographic information including: age, diagnosis (FASD group only), current gender identity, ethnicity, education level, employment status, and current income. The continuous variable (age) was reported as the mean value ± standard error of the mean (SEM). Categorical variables were reported as the percentage of the group followed by the *n* in parentheses. ^1^Mann Whitney *U* test, ^2^Fisher’s Exact Test, ^3^Chi Square Test.

Bolded *p*-values are significant; *post-hoc* testing – ** (*p* < 0.01): FASD > unexposed control; *(*p* < 0.05): FASD < unexposed control. FASD: Fetal Alcohol Spectrum Disorder.

### 2.3. Mental health and stress assessments

Mental health was assessed using the Beck Depression Inventory 2 (BDI-2; Pearson, Canada) and the Beck Anxiety Inventory (BAI; Pearson, Canada). The BDI is a 21-item, self-report questionnaire that assesses characteristic attitudes and symptoms of depression (Beck et al., 1961). A total score on the BDI ranging from 0 to 63 is obtained by adding the scores for each item. Total scores are classified as either: normal mood or minimal depression (score 0–13), mild depression (score 14–19), moderate depression (score 20–28), or severe depression (score 29–63). The BDI has been shown to have high reliability (Cronbach *α* = 0.92), with a 1-week test–retest reliability of *r* = 0.93 (Wang and Gorenstein, 2013), and to have the capacity to discriminate between individuals with and without depression, in a wide variety of populations (Osman et al., 2004; Wang and Gorenstein, 2013; Garcia-Batista et al., 2018; Toledano-Toledano and Contreras-Valdez, 2018). The BAI is a 21-item, self-report questionnaire assessing common somatic and cognitive symptoms of anxiety (Beck et al., 1988). A total score on the BAI ranging from 0–63 is obtained by adding the scores for each item. Total scores are classified as either: low anxiety (score 0–21), moderate anxiety (score 22–35), or high anxiety (score ≥ 36). The BAI has been reported to be highly reliable (Cronbach α =0.92), with a 1-week test–retest reliability of r = 0.75 (Beck et al., 1988; Fydrich et al., 1992). The BAI has also been shown to be able to discriminate between anxious and non-anxious diagnostic groups (Beck et al., 1988). The BDI and BAI were administered at baseline (pre-pandemic) during the in-person study visit and again during the pandemic, over the phone by trained study personnel. In addition to total scores for the BDI and BAI, a combined BDI/BAI score was generated by adding the BDI and BAI total scores, to examine mental health, in general.

### 2.4. Blood collection

Blood samples were drawn at the initial study visit for our CIFASD study (pre-pandemic/baseline) using EDTA-coated vacutainers, with separate tubes used to collect samples for cytokine and hematology analysis. Samples for cytokine analysis were placed on ice, centrifuged at 4°C, and plasma stored at −80°C. Samples for hematological analysis were kept at room temperature and analysis performed within 6 h of blood collection.

### 2.5. Hematology analysis and cytokine assays

Hematological analyses were performed using the Sysmex XN-550 automated hematology analyzer (Sysmex Canada, Mississauga, ON). Measures included a complete blood count (CBC), routine white blood cell differential, platelet counts, and hemoglobin level. Cytokine/chemokine and related inflammatory markers were measured in plasma samples using the Meso Scale Discovery (MSD) VLEX Human Biomarker 40-Plex kit (K15209D-1, MSD, Rockville, MD). Plates were read using a MESO QuickPlex SQ120 and data analyzed using the MSD Discovery Workbench software v.4.0. For the lower limit of detection and unabbreviated cytokine list, see Supplementary Table 2.

### 2.6. COVID-19 assessments

Self-report screeners including the Obsession with COVID-19 Scale (OCS), the Coronavirus Anxiety Scale (CAS) and the Coronavirus Reassurance Seeking Behaviors Scale (CRBS) were administered over the phone to assess COVID-related mental health problems. Specifically, the OCS identifies persistent and disturbed thinking related to COVID-19 (Lee, 2020), the CRBS measures reassurance-seeking behaviors associated with concerns over COVID-19 infection (Lee et al., 2020), and the CAS assesses dysfunctional anxiety related to the COVID-19 pandemic (Lee, 2020). Participants were also asked to self-report whether they had experienced a previous COVID-19 infection.

### 2.7. Statistical modeling

Analysis of demographic variables was performed using Mann–Whitney U test (age), Chi Square test (ethnicity), or the Fisher’s Exact test (gender, education level, employment status, current income, SES composite). The mental health assessments (BDI, BAI, BDI/BAI) and the COVID-19 assessments were analyzed by analysis of covariance (ANCOVA), having met the homogeneity of regression assumption. Repeated measures ANOVAs were also conducted to examine BDI, BAI, and BDI/BAI from baseline to pandemic. The rate of COVID-19 infection (self-report COVID-19 infection) was analyzed by Fisher’s Exact Test. Cytokine levels below the lower limit of detection of the assay were assigned a value of zero and outlier values were Winsorized (transformed to limit extreme outliers) (Tukey, 1962). The following cytokines were undetectable in >15% of the samples and were excluded from the analyses: Granulocyte-macrophage colony stimulating factor (GM-CSF; 78%), IL-17 (19%), IL-5 (75%), tumor necrosis factor-β (TNF-β; 19%), macrophage inflammatory protein-1α (MIP-1α; 44%), IL-13 (72.2%), IL-1β (63.9%), IL-2 (25%), IL-4 (75%). Cytokine values were non-normally distributed and were Blom transformed for statistical analyses. Hierarchical linear regression models were fit to examine the relationship between the SES composite and the COVID-19 assessments. To explore the relationship between cytokine levels (baseline) and mental health (baseline and pandemic), separate hierarchical linear regression models were fit for each cytokine. In addition, hierarchical linear regression was also performed to examine the relationship between baseline white blood cell counts and mental health (baseline and pandemic). For all hierarchical regression analyses, self-reported gender and age at the time of blood collection were controlled for in the model. *p* values were considered significant at *p* ≤ 0.05. Statistical analyses were conducted using IBM SPSS Statistics version 25.0.

## 3. Results

### 3.1. Sample characteristics

**Figure 1:**
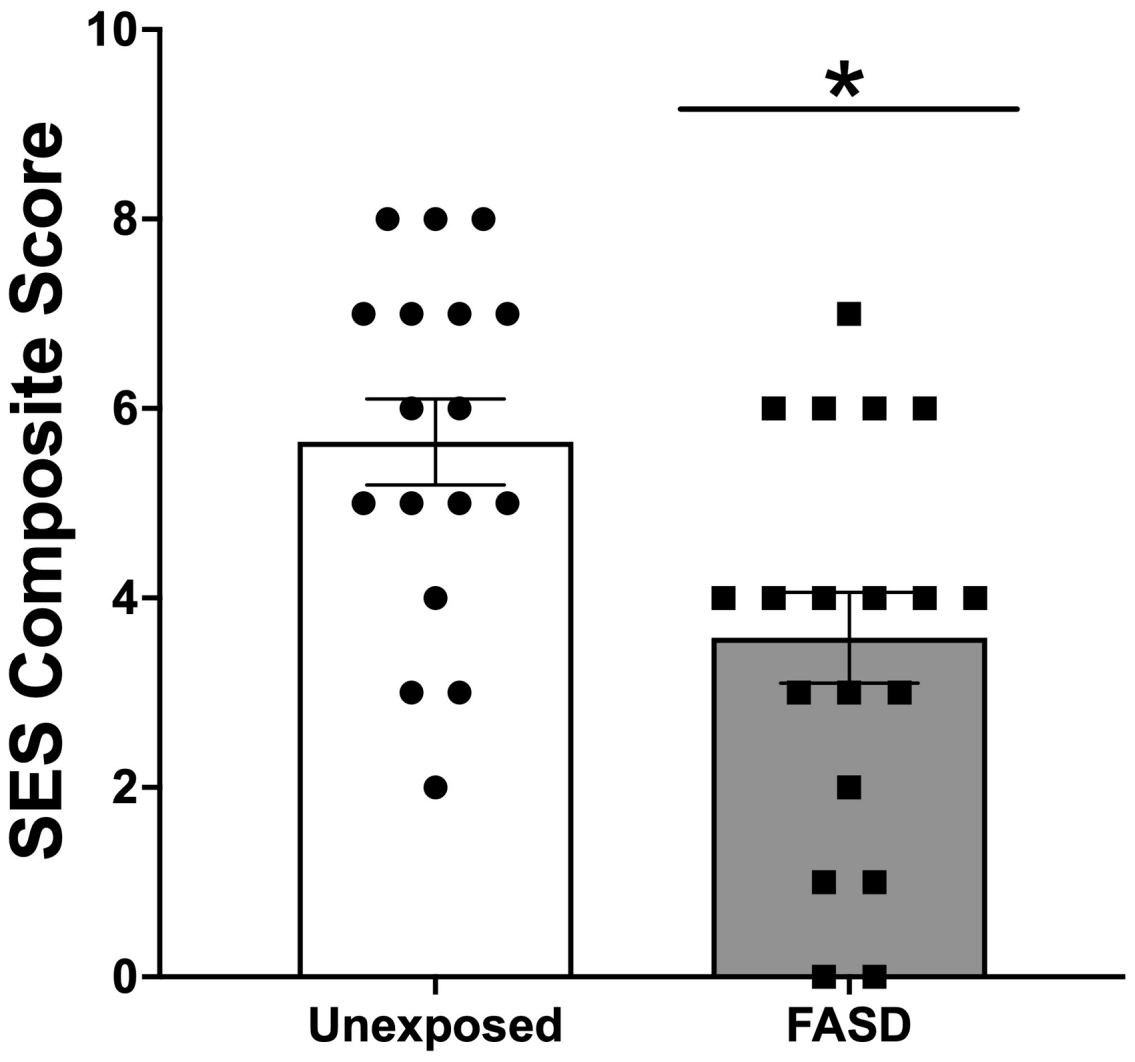
Socioeconomic status (SES) composite score generated from measures of education, income relative to the Canadian 2021 low income cut-off (adjusted for family size), occupation, and neighborhood. Higher scores indicated higher SES (and lower degree of socioeconomic disadvantage). SES was lower in adults with Fetal Alcohol Spectrum Disorder (FASD) compared to unexposed controls *p < 0.05*.*

There were no group differences in the age of study participants, education level, employment status, or current income (Table 1). For self-reported ethnicity [*X*^2^ (2, *n* = 36) = 9.24, *p =* 0.010], however, more adults with FASD reported their ethnicity as Indigenous, compared to unexposed controls (*p* = 0.004). In addition, unexposed controls reported higher rates of “other ethnicities” (e.g., South Asian/East Indian, Asian, other) compared to adults with FASD (*p* = 0.018) (Table 1). In the FASD group, 42.1% were diagnosed with Fetal Alcohol Syndrome (FAS), 10.5% were diagnosed with partial FAS (pFAS), and 5.3% were diagnosed with Alcohol Related Neurodevelopmental Disorder (ARND) (Table 1). In addition, 42.1% of adults with FASD could not recall their specific diagnosis; however, confirmation that the participant was, in fact, diagnosed with FASD was performed by research staff (through confirmation of details including diagnostic clinic and/or physician who performed the diagnosis). Finally, examination of the composite measure of SES showed that adults with FASD had lower SES (greater socioeconomic disadvantage), compared to unexposed adults [*X*^2^ (8, *n* = 36) = 15.71, *p* = 0.017; Figure 1].

A comparison of demographic variables, including age, gender, ethnicity, education level, employment status, current income, and baseline mental health (BDI, BAI) between the current COVID study participants (*n* = 36) and the overall CIFASD study sample recruited pre-pandemic (*n* = 72) revealed no differences between the two samples (Supplementary Table 3).

### 3.2. COVID-19 assessments

There were no differences in the self-reported rates of COVID-19 infection or scores on the CAS, the CRBS, or the OCS between adults with FASD and unexposed controls (Table 2). However, regression analysis revealed that lower SES was predictive of higher coronavirus anxiety on the CAS [∆*R^2^*: 0.289*, β*: −0.379, *F*(1, 32) = 5.18, *p* = 0.030] in the overall sample (no group differences). No associations were identified between SES and the CRBS or the OCS.

**Table 2:** COVID-19 assessments.

Variable	Unexposed control (*n* = 17)	FASD (*n* = 19)	Value of *p*
Do you think you have previously had a COVID-19 infection?	Yes: 23.5% (4)	Yes: 21.1% (4)	0.586^1^
Coronavirus Anxiety Scale (CAS)	2.0 ± 0.56	3.6 ± 1.1	0.215^2^
Coronavirus Reassurance Seeking Behaviors Scale (CRBS)	1.4 ± 0.7	2.3 ± 0.8	0.392^2^
Obsession with COVID-19 Scale (OCS)	2.0 ± 0.7	2.9 ± 1.0	0.471^2^

Participants were asked to report previous COVID-19 infection(s) and questionnaires were used to assess COVID-19-related anxieties/stressors. COVID-19 infection rates were reported as the percentage of the group followed by the *n* in parentheses. The COVID-19 scales are reported as the mean ± standard error of the mean (SEM). ^1^Fisher’s Exact Test, ^2^Analysis of covariance (ANCOVA). There were no differences between groups.

### 3.3. Mental health and immune function

**Figure 2:**
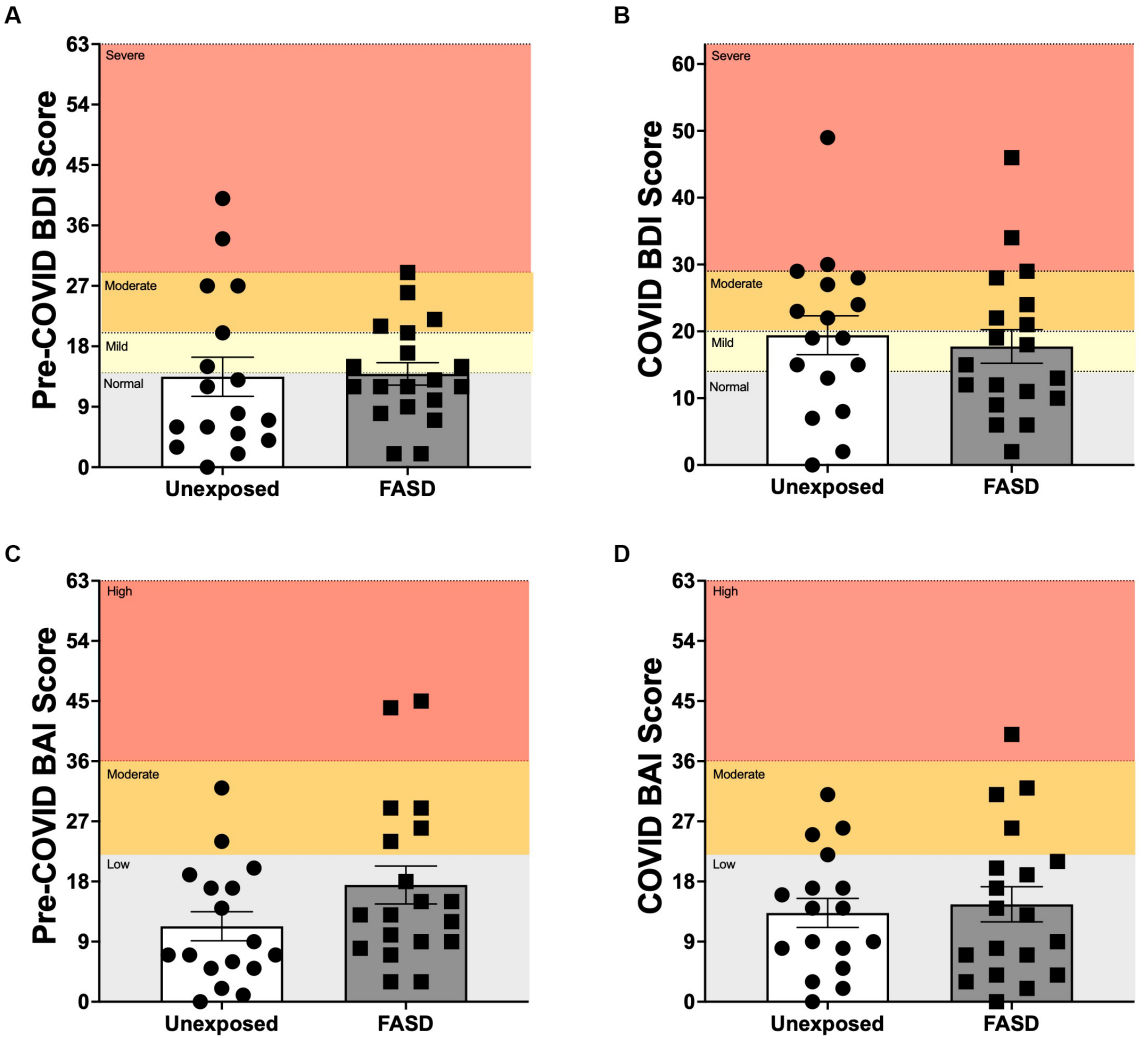
Beck Depression Inventory 2 (BDI), and Beck Anxiety Inventory (BAI) scores pre-COVID (baseline) and during the COVID-19 pandemic. **(A,B)** Normal: normal mood; Mild: mild mood disturbance; Moderate: moderate depression; Severe: severe depression. **(C,D)** Low: low anxiety; Moderate: moderate anxiety; High: high anxiety. There were no group differences between adults with FASD and unexposed adults on the BDI or BAI at baseline or during the pandemic, with the exception of a trend for higher pre-pandemic BAI in adults with FASD, compared to unexposed controls [*F*(1, 32) = 3.388, *p* = 0.075].

There were no differences between adults with FASD and unexposed adults on the BDI, BAI or overall mental health (BDI/BAI) either at baseline or during the pandemic, with the exception of a trend for increased pre-pandemic BAI in adults with FASD compared to unexposed controls [*F*(1, 32) = 3.388, *p* = 0.075; Figure 2]. In addition, repeated measures ANOVA of baseline to pandemic BDI and BAI similarly revealed no differences over time or by group.

To examine the relationship between immune function and mental health, hierarchical regression analyses were performed between baseline white blood cell counts and overall mental health (BDI/BAI). Regression analyses identified a positive association between neutrophil counts and pre-pandemic BDI/BAI score in unexposed controls [∆*R^2^*: 0.307*, β*: 0.575, *F*(1, 12) = 5.65, *p* = 0.035] but not in adults with FASD. Similarly, a positive association was also identified between baseline neutrophil counts and pandemic BDI/BAI scores again in unexposed adults only [∆*R^2^*: 0.268*, β*: 0.548, *F*(1, 12) = 4.49, *p* = 0.05]. No associations between lymphocyte and monocyte counts and baseline or pandemic BDI/BAI scores were identified.

To further explore the link between immune function and mental health, hierarchical regression analyses were performed between baseline cytokine levels and BDI/BAI scores (baseline and pandemic) (Table 3). Interestingly, baseline cytokine levels failed to predict current (pre-pandemic) BDI/BAI scores. However, associations between baseline cytokines and pandemic BDI/BAI scores were identified. In unexposed controls, there was a negative association between pre-pandemic IFN-ɣ and pandemic BDI/BAI scores [∆*R^2^*: 0.411*, β*: −0.664, *F*(1, 12) = 8.59, *p* = 0.013]. In adults with FASD, there were positive associations between baseline levels of IL-12p70 [∆*R^2^*: 0.231*, β*: 0.471, *F*(1, 15) = 4.53, *p* = 0.050], IL-8 [∆*R^2^*: 0.368*, β*: 0.738, *F*(1, 15) = 8.76, *p* = 0.010], soluble intercellular adhesion molecule-1 (sICAM-1) [∆*R^2^*: 0.273*, β*: 0.781, *F*(1, 15) = 5.65, *p* = 0.031], and soluble vascular cell adhesion molecule-1 (sVCAM-1) [∆*R^2^*: 0.235, *β*: 0.587, *F*(1, 15) = 4.62, *p* = 0.048] and pandemic BDI/BAI scores (see Supplementary Tables 4A,B for non-significant results).

**Table 3:** Summary of hierarchical regression analyses between pre-pandemic cytokine levels and pandemic BDI/BAI scores.

	IFN-ɣ	IL-12p70	IL-8	sICAM-1	sVCAM-1
Unexposed control (*n* = 17)					
∆*R^2^*	0.411	n.s	n.s	n.s	n.s
*β*	−0.664				
*F*	8.59				
*p*	0.013				
FASD (*n* = 19)					
∆*R^2^*	n.s	0.231	0.368	0.273	0.235
*β*		0.471	0.738	0.781	0.587
*F*		4.53	8.76	5.65	4.62
*p*		0.050	0.010	0.031	0.048

Hierarchical regression analyses were performed between baseline cytokine levels and mental health scores (combined Beck Depression Inventory [BDI] and Beck Anxiety Inventory [BAI] score) during the pandemic. For unexposed controls, there was a negative association between pre-pandemic IFN-ɣ and pandemic BDI/BAI scores. For adults with FASD, there were positive associations between baseline levels of interleukin (IL)-12p70, IL-8, soluble intercellular adhesion molecule-1 (sICAM-1), and soluble vascular cell adhesion molecule-1 (sVCAM-1) and pandemic BDI/BAI scores. Unexposed control: df: 1, 12; FASD: df: 1, 15; n.s. non-significant.

## 4. Discussion

The goal of the current study was to examine the impact of the COVID-19 pandemic on mental health in adults with FASD and to probe for inflammation-to-mood associations. Overall, while there were no differences between adults with FASD and unexposed controls on COVID-19 assessments, lower SES was predictive of higher coronavirus anxiety scores on the CAS in the overall study sample. Perhaps surprisingly, there were no differences in depression and anxiety measures at baseline or during the pandemic between adults with FASD and unexposed controls. However, for unexposed controls but *not* adults with FASD, we identified a positive association between baseline neutrophil counts and both baseline and pandemic mental health scores. Moreover, we found differential associations between baseline cytokines and mental health scores during the pandemic in unexposed controls versus adults with FASD. Specifically, a negative association between baseline IFN-ɣ and pandemic mental health scores was observed in controls while positive associations between baseline IL-12p70, IL-8, sICAM-1, and sVCAM-1 and pandemic mental health scores were identified in adults with FASD. Taken together, these data highlight that while mental health scores were not different between adults with FASD and unexposed adults during the pandemic, differential inflammation-to-mood relationships were identified, suggesting a possible role for altered immune function in these differential mental health outcomes. Future work is needed to explore the long-terms impacts of the COVID-19 pandemic on these associations.

The observed link between SES and anxiety around coronavirus—specifically lower SES being predictive of higher coronavirus anxiety scores on the CAS—is in line with other studies in which financial difficulties, unemployment, and lower levels of education were correlated with poor mental health during the pandemic (Smith et al., 2020; Wang et al., 2020; Nagasu et al., 2021). Importantly, while we replicated these previous findings, demonstrating a relationship between SES and anxiety around coronavirus in our full sample, adults with FASD make up a higher proportion of those with low SES and thus may be at disproportionately high risk of anxiety related to coronavirus. Similar findings have been reported in other NDDs where, for example, individuals with ASD may experience a higher rate of confusion around the pandemic, which in turn impacts their mental health (Oomen et al., 2021). While not explicitly examined, many of our participants with FASD reported confusion around public health measures and repeated misinformation related to coronavirus spread and symptoms of illness. Moving forward, it will be important that messaging around societal crises takes into account the specific needs of individuals with NDDs, including FASD.

The current study did not identify differences in depression or anxiety scores between adults with FASD and unexposed controls, with the exception of a trend for higher anxiety scores at baseline in adults with FASD. This was perhaps surprising as adults with FASD were predicted to be at higher risk of mental health problems during the pandemic as a result of risk factors including lower SES (May and Gossage, 2011; Kwong et al., 2021) and higher rates of existing mental health problems (Famy et al., 1998; Pei et al., 2011; Neelam et al., 2021). However, it has been recognized that the pandemic afforded some positive effects for individuals with NDDs, including reduced sensory and social overloading (Oomen et al., 2021). As FASD is associated with impaired social abilities (Thomas et al., 1998; Tsang et al., 2016), it stands to reason that the reduction in social demands during the pandemic could have had some beneficial impact for adults with FASD. Clearly, however, the pandemic also impacted the availability of social supports and programming that many adults with FASD rely on, which underscores the need for future work to examine the long-term impacts of the pandemic on mental health in this population, as well as underlying factors contributing to these impacts.

The association between immune function and mental health has been well established (Raison et al., 2006) such that at the extreme end, major depressive disorder (MDD) is associated with a proinflammatory bias and impaired adaptive immune functioning (Irwin et al., 1990; Leday et al., 2018; Beurel et al., 2020). And neutrophils, being the most abundant leukocyte and important cytokine secretors, are well-positioned to modulate the inflammation-to-mood relationship. Thus, the identification of a positive association between baseline neutrophil counts and mental health scores (baseline and pandemic) was expected. Additionally, previous work has shown positive correlations between depression scores and neutrophil counts during the pandemic (Li et al., 2021). However, in the current study, the association between neutrophils and mental health scores was only detected in unexposed controls. By contrast, the lack of a relationship between mental health status and neutrophil counts in adults with FASD may represent altered immune-to-brain communication in these adults, or differential immune cell and/or cytokine responses driving inflammation-to-mood associations.

The current study also identified a link between inflammatory mediators and mood in individuals with FASD, consisting of positive associations between baseline IL-12, IL-8, sICAM-1, and sVCAM-1 and pandemic mental health scores. IL-12 in particular, has been strongly linked to depression in the literature. In a meta-analysis involving 82 studies, IL-12 was shown to be higher in individuals with MDD (Kohler et al., 2018). IL-8, which can have both pro- and anti-inflammatory functions, has been less consistently identified in individuals with mood disorders (Kohler et al., 2018); however, elevated IL-8 has been suggested as an acute biomarker of a variety of psychiatric conditions (Gariup et al., 2015), as well as other chronic conditions (Shahzad et al., 2010). Moreover, cell adhesion molecules (CAMs), including ICAM-1 and VCAM-1, transmembrane adhesion proteins expressed predominantly in endothelial cells, are critical to the integrity of the blood brain barrier, with dysregulation linked to immune-mediated trafficking to the central nervous system and neuroinflammation (Muller et al., 1999; Sheikh et al., 2023). Soluble isoforms (e.g., sICAM-1, sVCAM-1) reflect an indirect plasma measure, with increases in sICAM-1 shown in conditions including depression and dementia, as well as aging [reviewed in Muller (2019)] and increases in sVCAM-1 identified in inflammatory disorders (Park and Jeen, 2018). Thus, in the current study, higher levels of sICAM-1 and sVCAM-1 may be contributing to neuroinflammatory processes in individuals with FASD and subsequently, driving increasing scores on mental health assessments. By contrast only a single association between baseline cytokines and mental health scores was identified in unexposed controls – a negative association between baseline IFN-ɣ and pandemic mental health scores. It is possible that the stress of the pandemic, acting on a dysregulated immune system in adults with FASD, may have had a bigger impact on the inflammatory environment and subsequently mood, than it did in unexposed controls.

Finally, it is important to consider possible limitations of the current study. First, re-examining mental health in a vulnerable population during a global pandemic was challenging and it is possible that the study sample reflected some degree of selection bias. For example, it stands to reason that the study sample could have been biased towards more resilient individuals, potentially explaining the lack of a detectable impact of the pandemic on mental health outcomes. A comparison of pre-pandemic mental health scores between current study participants and the full cohort, however, showed no differences, suggesting that at least with regards to mental health, the current cohort was representative of the overall sample. However, it is possible that despite no baseline differences in mental health scores, individuals who did not participate in the current study could have been experiencing more life difficulties/stressors during the pandemic. To this end, it was more difficult to re-recruit adults with FASD from our overall study sample, as compared to controls, resulting in the small sample size, which must be acknowledged. More research in this area, and research involving other populations of adults with FASD will be needed to confirm the generalizability of the current findings. Finally, it is important to consider the time frame in which this study was completed. Major closures and establishment of public health measures aimed at decreasing the spread of COVID-19 were implemented in Canada beginning in March 2020. This study began 10 months later and while this timing coincided with the biggest peak to that point in time in COVID-19 infections in BC, “pandemic fatigue,” referring to waning of precautionary behaviors, was also emerging (Brankston et al., 2022), which may also have impacted mental health outcomes.

In conclusion, our findings are important as there has been minimal research examining the impact of the COVID-19 pandemic on individuals with FASD and, to our knowledge, this is the first study to investigate outcomes in adults with FASD. While it may be too soon to predict the impact this pandemic will have on long-term mental health, our research suggests that the pandemic differentially impacted inflammation-to-mood relationships between adults with FASD and unexposed controls and highlights the need for longitudinal research in this area.

## Data availability statement

The raw data supporting the conclusions of this article will be made available by the authors, without undue reservation.

## Ethics statement

The studies involving human participants were reviewed and approved by the University of British Columbia Children’s and Women’s Research Ethics Board. The patients/participants provided their written informed consent to participate in this study.

## Author contributions

TSB contributed to the conception and design of the study and funding acquisition, performed the statistical analyses, and wrote the first draft of the manuscript. AC contributed to testing study participants, data management, and project administration. PJH contributed to testing study participants and assisted with the ethics submission. LE assisted with the ethics submission and data and study administration. CR contributed to the conception and design of the study, interpretation of the data, and funding acquisition. JW contributed to the conception and design of the study and funding acquisition. All authors contributed to manuscript revisions and approved the submitted version.

## Funding

All or part of this work was done in conjunction with the Collaborative Initiative on Fetal Alcohol Spectrum Disorders (CIFASD), which is funded by grants from the National Institute on Alcohol Abuse and Alcoholism (NIAAA). Additional information about CIFASD can be found at www.cifasd.org. Funded by NIH/NIAAA U01 AA026101 to JW and NIH/NIAAA R01 AA022460 to JW, CR, and TSB.

## Conflict of interest

The authors declare that the research was conducted in the absence of any commercial or financial relationships that could be construed as a potential conflict of interest.

## Publisher’s note

All claims expressed in this article are solely those of the authors and do not necessarily represent those of their affiliated organizations, or those of the publisher, the editors and the reviewers. Any product that may be evaluated in this article, or claim that may be made by its manufacturer, is not guaranteed or endorsed by the publisher.
