# Supplementary material for: Impact of the COVID-19 pandemic on adults with Fetal Alcohol Spectrum Disorder: linking immune function to mental health status
Source: Front Neurosci. 2023 Jul 19;17:1214100. doi: 10.3389/fnins.2023.1214100 (PMC10394466; doi:10.3389/fnins.2023.1214100)
Supplement: Supplementary file 1 [file Data_Sheet_1.docx]

**Supplementary Table 1**: Socioeconomic Status (SES) Composite Variable

| **Variable** | **Category** | **Score** |
| --- | --- | --- |
| Education | Post-high school education | 2 |
|  | High school graduate | 1 |
|  | Incomplete high school | 0 |
| Income relative to the Canadian 2021 low income cut-off (LICO; adjusted for family size) | >150% of LICO | 2 |
|  | 100 – 150% of LICO | 1 |
|  | < LICO | 0 |
| Occupation | Employed (non-manual) | 2 |
|  | Employed (manual/part-time) | 1 |
|  | Unemployed | 0 |
| Neighborhood – Safe to walk alone at night | Safe | 2 |
|  | Somewhat safe | 1 |
|  | Mostly unsafe | 0 |

Summary of the variables used to produce the SES composite. Each variable was given a score from 0 (most disadvantage) to 2 (least disadvantage), with scores summed to produce a total score ranging from 0 – 8.

**Supplementary Table 2**: Cytokine assay lower limits of detection (LLOD)

| **Cytokine** | | **LLOD (pg/mL)** |
| --- | --- | --- |
| C-reactive protein | CRP | 4.93 |
| Eotaxin | Eotaxin  (or CCL11) | 23.70 |
| Eotaxin-3 | Eotaxin-3  (or CCL26 or MIP-4α) | 1.98 |
| Fms-like tyrosine kinase/vascular endothelial growth factor receptor 1 | sFlt-1/VEGFR1 | 0.913 |
| Basic fibroblast growth factor | bFGF | 0.185 |
| Granulocyte-macrophage colony stimulating factor | GM-CSF | 0.119 |
| Intercellular adhesion molecule-1 | sICAM-1 | 3.35 |
| Interferon-γ | IFN-γ | 0.308 |
| Interleukin-1α | IL-1α | 0.472 |
| Interleukin-1β | IL-1β | 0.043 |
| Interleukin-10 | IL-10 | 0.028 |
| Interleukin-12p70 | IL-12p70 | 0.044 |
| Interleukin-12/Interleukin-23 p40 | IL-12/IL-23p40 | 0.266 |
| Interleukin-13 | IL-13 | 0.347 |
| Interleukin-15 | IL-15 | 0.108 |
| Interleukin-16 | IL-16 | 0.828 |
| Interleukin-17A | IL-17A | 0.960 |
| Interleukin-2 | IL-2 | 0.042 |
| Interleukin-4 | IL-4 | 0.026 |
| Interleukin-5 | IL-5 | 0.090 |
| Interleukin-6 | IL-6 | 0.080 |
| Interleukin-7 | IL-7 | 0.097 |
| Interleukin-8 | IL-8  (or CXCL8) | 0.526 |
| Interferon gamma-induced protein | IP-10  (or CXCL10) | 2.83 |
| Monocyte chemotactic protein 1 | MCP-1  (or CCL2) | 1.27 |
| Monocyte chemotactic protein 4 | MCP-4  (or CCL13) | 11.10 |
| Macrophage-derived chemokine | MDC  (or CCL22) | 18.10 |
| Macrophage inflammatory protein 1α | MIP-1α  (or CCL3) | 13.10 |
| Macrophage inflammatory protein 1β | MIP-1β  (or CCL4) | 3.12 |
| Placental growth factor | PlGF | 0.272 |
| Serum amyloid A | SAA | 20.9 |
| Thymus and activation regulated chemokine | TARC  (or CCL17) | 3.40 |
| Tyrosine kinase-2 | Tie-2 | 38.00 |
| Tumor necrosis factor α | TNF-α | 0.050 |
| Tumor necrosis factor β | TNF-β | 0.080 |
| Vascular cell adhesion molecule-1 | VCAM-1 | 9.76 |
| Vascular endothelial growth factor-A | VEGF-A | 0.965 |
| Vascular endothelial growth factor-C | VEGF-C | 13.40 |
| Vascular endothelial growth factor-D | VEGF-D | 5.79 |

Table showing the lower limit of detection (LLOD) for each analyte (cytokine/chemokine/related factors) and the unabbreviated cytokine names/alternative names.

**Supplementary Table 3**: Demographic variable comparison between full sample (original CIFASD study) and current COVID study sample

| **Variable** | | **Full sample**  **(n=72)** | **COVID sample**  **(n=36)** | ***P* value** |
| --- | --- | --- | --- | --- |
| Age (years) | | 38.2 ± 1.3 | 36.7 ± 1.9 | 0.434^1^ |
| Current Gender Identity | Man | 26.4% (19) | 22.2% (8) | 0.764^2^ |
|  | Woman | 72.2% (52) | 77.8%(28) |  |
|  | Transgender | 1.4% (1) | 0% (0) |  |
| Ethnicity | Indigenous | 47.2% (34) | 38.9% (14) | 0.600^3^ |
|  | White | 30.6% (22) | 30.6% (11) |  |
|  | Other ethnicities | 22.2% (16) | 30.6% (11) |  |
| Education Level | Incomplete high school | 25.0% (18) | 19.4% (7) | 0.889^3^ |
|  | Graduated high school | 22.2% (16) | 19.4% (7) |  |
|  | Partial college | 13.9 (10) | 13.9% (5) |  |
|  | College or University graduate | 19.4% (14) | 19.4 (7) |  |
|  | Graduate professional training | 19.4% (14) | 27.8% (10) |  |
| Employment Status | Employed | 44.4% (32) | 55.6% (20) | 0.765^2^ |
|  | Full-time student (or vocational training) | 5.6% (4) | 2.8% (1) |  |
|  | Volunteer work | 9.7% (7) | 5.6% (2) |  |
|  | Unemployed | 38.9% (28) | 33.3% (12) |  |
|  | Missing | 1.4% (1) | 2.8% (1) |  |
| Current Income | <$20,000 | 43.1% (31) | 41.7% (15) | 0.906^2^ |
|  | $20,000 - $49,999 | 29.2% (21) | 25.0% (9) |  |
|  | $50,000 - $99,999 | 6.9% (5) | 11.1% (4) |  |
|  | >$100,000 | 13.9% (10) | 16.7% (6) |  |
|  | Missing | 7.0% (5) | 5.6% (2) |  |
| Baseline mental health | Beck Depression Inventory 2 (BDI) scores | 14.2 ± 1.3 | 13.8 ± 1.6 | 0.978^1^ |
|  | Beck Anxiety Inventory (BAI) scores | 14.7 ± 1.5 | 14.6 ± 1.9 | 0.640^1^ |

Key demographic variables were compared between the original CIFASD sample and the COVID study sample (subset of the original sample). There were no differences between the two samples on any of the demographic variables examined.

^1^Mann Whitney U test; ^2^Fisher’s Exact test, ^3^Chi Square test

FASD: Fetal Alcohol Spectrum Disorder (FASD)

**Supplementary Table 4A**: Summary of hierarchical regression analyses between pre-pandemic cytokine levels and pandemic BDI/BAI scores

|  | **IL-12** | **IL-15** | **IL-16** | **IL-1α** | **VEGF** | **Eotaxin** | **Eotaxin-3** | **IP-10** | **MCP-4** | **MDC** |
| --- | --- | --- | --- | --- | --- | --- | --- | --- | --- | --- |
| **Unexposed control (*n*=17)** | | | | | |  |  |  |  |  |
| ∆*R^2^* | 0.015 | 0.152 | 0.003 | 0.083 | 0.036 | 0.009 | 0.037 | 0.005 | 0.0001 | 0.009 |
| β | 0.123 | 0.108 | -0.054 | 0.289 | 0.191 | -0.096 | -0.194 | 0.074 | 0.011 | 0.096 |
| *F* | 0.219 | 0.152 | 0.058 | 1.723 | 0.566 | 0.264 | 0.818 | 0.113 | 0.002 | 0.161 |
| *p* | 0.648 | 0.703 | 0.814 | 0.214 | 0.466 | 0.617 | 0.383 | 0.743 | 0.962 | 0.695 |
| **FASD (*n*=19)** | | | | | |  |  |  |  |  |
| ∆*R^2^* | 0.005 | 0.041 | 0.040 | 0.021 | 0.002 | 0.464 | 0.013 | 0.254 | 0.001 | 0.084 |
| β | -0.072 | 0.202 | 0.201 | -0.145 | 0.041 | -0.144 | 0.112 | 0.338 | 0.026 | 0.290 |
| *F* | 0.090 | 0.671 | 0.629 | 0.615 | 0.029 | 0.606 | 0.234 | 2.713 | 0.012 | 1.441 |
| *p* | 0.768 | 0.426 | 0.441 | 0.445 | 0.868 | 0.449 | 0.636 | 0.120 | 0.916 | 0.249 |

**Supplementary Table 4B**: Summary of hierarchical regression analyses between pre-pandemic cytokine levels and pandemic BDI/BAI scores

|  | **TARC** | **IL-10** | **IL-6** | **TNF-α** | **Tie-2** | **VEGFC** | **VEGFD** | **bFGF** | **sFlt-1** | **CRP** | **SAA** |
| --- | --- | --- | --- | --- | --- | --- | --- | --- | --- | --- | --- |
| **Unexposed control (*n*=17)** | | | | | |  |  |  |  |  |  |
| ∆*R^2^* | 0.001 | 0.121 | 0.0004 | 0.015 | 0.209 | 0.00003 | 0.034 | 0.060 | 0.009 | 0.001 | 0.004 |
| β | 0.036 | -0.208 | 0.019 | -0.123 | 0.460 | 0.005 | 0.187 | 0.246 | 0.097 | -0.035 | -0.060 |
| *F* | 0.027 | 0.609 | 0.006 | 0.261 | 3.507 | 0.0003 | 0.575 | 0.798 | 0.126 | 0.015 | 0.047 |
| *p* | 0.871 | 0.450 | 0.940 | 0.619 | 0.086 | 0.986 | 0.463 | 0.389 | 0.729 | 0.906 | 0.832 |
| **FASD (*n*=19)** | | | | | |  |  |  |  |  |  |
| ∆*R^2^* | 0.135 | 0.002 | 0.004 | 0.056 | 0.002 | 0.133 | 0.004 | 0.070 | 0.055 | 0.017 | 0.049 |
| β | -0.131 | 0.047 | 0.061 | 0.237 | -0.043 | 0.365 | 0.065 | 0.278 | 0.234 | 0.129 | 0.222 |
| *F* | 2.485 | 0.035 | 0.059 | 0.943 | 0.030 | 2.369 | 0.066 | 1.304 | 1.075 | 0.487 | 1.068 |
| *p* | 0.136 | 0.855 | 0.811 | 0.347 | 0.866 | 0.145 | 0.800 | 0.272 | 0.316 | 0.492 | 0.318 |

Summary of the non-significant hierarchical regression results. Hierarchical regression analyses were performed between baseline cytokine levels and mental health scores (combined Beck Depression Inventory [BDI] and Beck Anxiety Inventory [BAI] score) during the pandemic.

Unexposed control: df: 1, 12; FASD: df: 1, 15

FASD: Fetal Alcohol Spectrum Disorder
